# Diverging Mortality Trends by Educational Attainment in the US

**DOI:** 10.1001/jamahealthforum.2025.1647

**Published:** 2025-06-13

**Authors:** Eugenio Paglino, Elizabeth Wrigley-Field, Andrew C. Stokes

**Affiliations:** 1 Helsinki Institute for Demography and Population Health, University of Helsinki, Helsinki, Finland; 2 Max Planck–University of Helsinki Center for Social Inequalities in Population Health, Helsinki, Finland; 3 Department of Sociology, University of Minnesota, Minneapolis; 4 Minnesota Population Center and Institute for Social Research and Data Innovation, University of Minnesota, Minneapolis; 5 Department of Global Health, Boston University School of Public Health, Boston, Massachusetts

## Abstract

This cross-sectional study examines trends in US mortality rates by sex and educational attainment before, during, and after the COVID-19 pandemic.

## Introduction

After decades of improvement, US mortality rate decreases have stagnated or reversed since 2010.^1,2,3^ Research suggests these trends are greatest among adults with lower educational attainment.^4^ We describe differences in mortality rates before, during, and after the COVID-19 pandemic by estimating (across educational attainment and sex) how many more deaths occurred between 2011 and 2023 than would have been expected based on 2006-2010 trends.

## Methods

This cross-sectional study did not involve human participants and was deemed exempt by the Boston University Institutional Review Board. Informed consent was waived. We followed the STROBE guideline.

We obtained US death and population counts by age (≥35 years), sex, year, and education from the National Vital Statistics System and the 1-year American Community Survey Public Use Microdata Sample. Educational attainment was categorized as having a bachelor’s degree or higher (college graduate) vs having less than a bachelor’s degree (noncollege graduate).^4^ We computed age- and cause-specific mortality rates by sex and educational attainment. We used National Health Interview Survey Linked Mortality File (1986-2018) estimates to correct for education misreporting on death certificates.^5^ We modeled cause-specific age-standardized mortality rates using generalized additive models. We used 2006-2010 as a baseline and forecasted mortality rates for the prepandemic (2011-2019), pandemic (2020-2022), and postpandemic (2023) periods. We computed excess mortality as the difference between observed and expected mortality. To quantify the uncertainty in the estimates, we report 80% prediction intervals (PIs). R, version 4.1.1 (R Project for Statistical Computing), was used for data analysis. Additional details are provided in Supplement 1.

## Results

We analyzed 47 545 611 US deaths for 2006 to 2023. Mortality was markedly higher during the prepandemic, pandemic, and postpandemic periods (2011-2023) than would have been expected based on 2006-2010 trends for both male and female noncollege graduates (Figure). Excess deaths also occurred during 2011 to 2023 for male college graduates but to a lesser extent. Although COVID-19 was a leading cause of excess deaths, much of the increase in excess deaths over 2020 to 2023 was associated with increases in deaths from circulatory diseases and diabetes.

**Figure.**
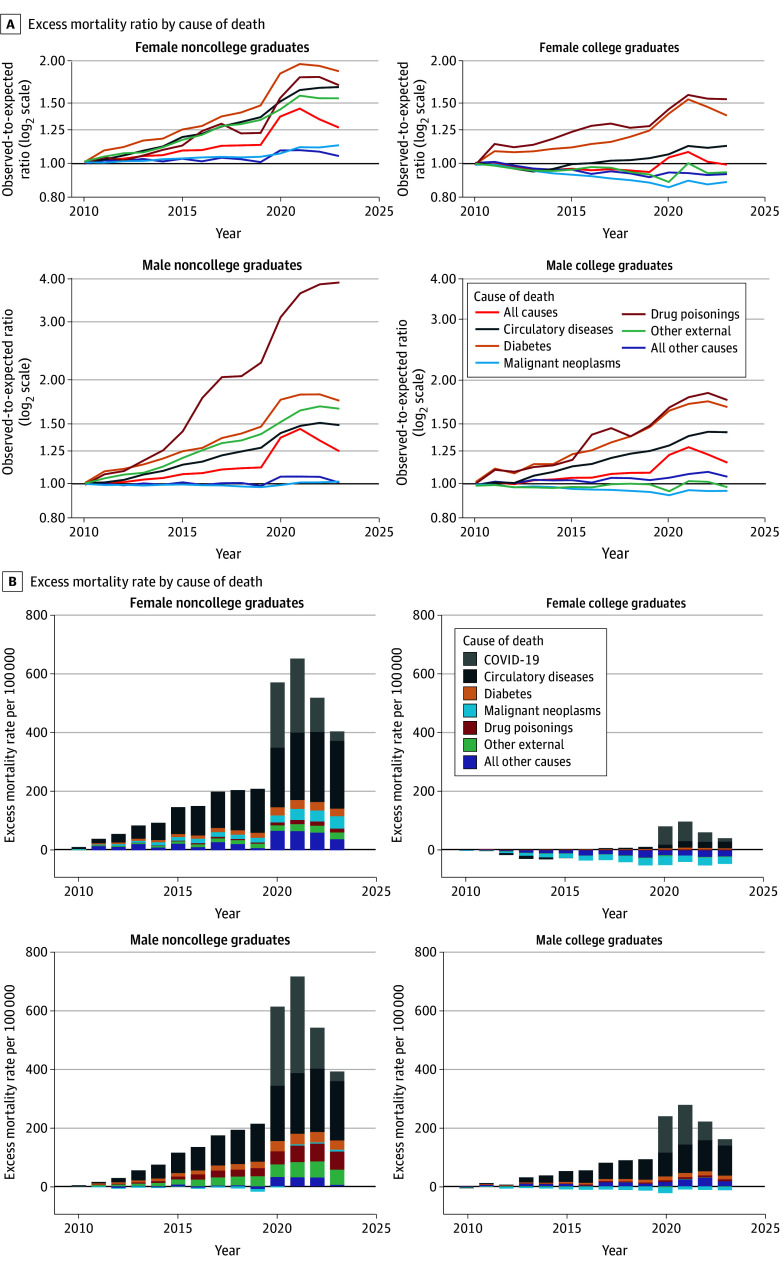
Cause-Specific Excess Mortality Ratios and Rates by Sex and Educational Attainment Among Individuals Aged 35 Years or Older, 2011-2023 A and B, Excess mortality ratios and rates, respectively. Excess mortality rates were estimated as the difference between observed and expected age-standardized mortality rates. Expected rates were forecasted from the baseline period (2006-2010). Educational attainment was categorized as having a bachelor’s degree or higher (college graduate) vs having less than a bachelor’s degree (noncollege graduate). In B, the excess mortality rate for each year is the total height of the bar.

An estimated 525 505 (80% PI, 437 312-609 298) more deaths occurred in 2023 than would have been expected based on 2006-2010 mortality trends. Among noncollege graduates, mortality was 26% higher (80% PI, 21%-32%) in 2023 compared with what would have been expected based on 2006-2010 trends, equaling 481 211 (80% PI, 401 771-564 855) excess deaths (Table). In contrast, mortality was only 8% higher (80% PI, 3%-13%) among college graduates in 2023 compared with baseline, equaling 44 294 (80% PI, 19 838-69 239) excess deaths. Among both male and female noncollege graduates, circulatory diseases were the leading cause of excess deaths in 2023 (51.5% vs 56.9%, respectively). In contrast, among male vs female noncollege graduates, 15.8% vs 3.4% of excess deaths were attributable to drug poisonings, and 13.2% vs 5.7% of excess deaths were due to other external causes, respectively.

**Table.** Observed, Counterfactual, and Excess US Deaths Among Individuals Aged 35 Years or Older, 2023^a^

Cause of death, by sex and educational attainment	No. of observed deaths	No. of counterfactual deaths (80% PI)	Ratio of observed to counterfactual deaths (80% PI)	No. of excess deaths (80% PI)	Percentage of total^b^
**Males**					
<Bachelor’s degree					
All causes	1 179 368	948 957 (911 653-987 353)	1.24 (1.19-1.29)	230 410 (192 015-267 715)	100
Circulatory diseases	367 528	248 882 (234 914-263 372)	1.48 (1.40-1.56)	118 645 (104 155-132 613)	51.5
COVID-19	19 239	0	NA	19 239	8.3
Diabetes	43 971	25 326 (23 677-27 001)	1.74 (1.63-1.86)	18 645 (16 971-20 294)	8.1
Malignant neoplasms	242 629	239 198 (231 425-247 399)	1.02 (0.98-1.05)	3431 (−4770 to 11 203)	1.5
Drug poisonings	49 193	12 890 (12 234-13 595)	3.82 (3.62-4.02)	36 303 (35 598-36 959)	15.8
Other external	77 034	46 673 (39 262-54 597)	1.68 (1.41-1.96)	30 360 (22 437-37 771)	13.2
All other causes	379 775	375 988 (347 260-402 402)	1.01 (0.94-1.09)	3787 (−22 627 to 32 515)	1.6
≥Bachelor’s degree					
All causes	348 458	302 367 (290 253-315 047)	1.15 (1.11-1.20)	46 091 (33 411-58 205)	100
Circulatory diseases	110 314	78 189 (74 256-82 060)	1.41 (1.34-1.49)	32 125 (28 254-36 058)	69.7
COVID-19	6621	0	NA	6621	14.4
Diabetes	10 055	6066 (5517-6648)	1.67 (1.51-1.82)	3989 (3407-4538)	8.7
Malignant neoplasms	77 658	81 423 (78 132-84 807)	0.95 (0.92-0.99)	−3765 (−7149 to −474)	−8.2
Drug poisonings	4296	2451 (2112-2833)	1.78 (1.52-2.03)	1846 (1463-2185)	4.0
Other external	21 037	21 544 (19 591-23 587)	0.98 (0.89-1.07)	−507 (−2550 to 1446)	−1.1
All other causes	118 477	112 695 (100 999-125 965)	1.06 (0.94-1.17)	5782 (−7488 to 17 478)	12.5
Total	1 527 826	1 251 325 (1 210 109-1 291 413)	1.22 (1.18-1.26)	276 501 (236 413-317 717)	100
**Females**					
<Bachelor’s degree					
All causes	1 175 505	924 704 (854 937-996 164)	1.28 (1.18-1.37)	250 801 (179 340-320 568)	100
Circulatory diseases	358 918	216 110 (198 738-234 642)	1.67 (1.53-1.81)	142 809 (124 276-160 180)	56.9
COVID-19	19 850	0	NA	19 850	7.9
Diabetes	34 896	18 823 (16 506-21 004)	1.87 (1.66-2.11)	16 073 (13 892-18 391)	6.4
Malignant neoplasms	225 506	200 745 (184 290-217 440)	1.13 (1.04-1.22)	24 761 (8065-41 216)	9.9
Drug poisonings	20 466	11 982 (9234-15 213)	1.77 (1.35-2.22)	8484 (5254-11 232)	3.4
Other external	39 927	25 716 (22 709-29 063)	1.57 (1.37-1.76)	14 211 (10 864-17 218)	5.7
All other causes	475 941	451 328 (402 081-501 008)	1.06 (0.95-1.18)	24 614 (−25 067 to 73 860)	9.8
≥Bachelor’s degree					
All causes	256 670	258 468 (238 421-278 793)	1.00 (0.92-1.08)	−1797 (−22 123 to 18 249)	NA
Circulatory diseases	71 604	63 361 (52 689-74 346)	1.15 (0.96-1.36)	8243 (−2742 to 18 915)	NA
COVID-19	3953	0	NA	3953	NA
Diabetes	5455	3941 (3359-4561)	1.40 (1.20-1.62)	1514 (894-2096)	NA
Malignant neoplasms	62 949	71 166 (65 290-77 387)	0.89 (0.81-0.96)	−8217 (−14 438 to −2341)	NA
Drug poisonings	2396	1600 (941-2321)	1.71 (1.03-2.55)	796 (75-1456)	NA
Other external	10 259	10 916 (8713-13 435)	0.97 (0.76-1.18)	−657 (−3176 to 1546)	NA
All other causes	100 053	107 482 (95 485-119 804)	0.94 (0.84-1.05)	−7429 (−19 751 to 4568)	NA
Total	1 432 175	1 183 172 (1 111 250-1 256 585)	1.21 (1.14-1.29)	249 003 (175 590-320 925)	100
**All-cause deaths for males and females**					
<Bachelor’s degree	2 354 872	1 873 662 (1 790 017-1 953 102)	1.26 (1.21-1.32)	481 211 (401 771-564 855)	100
≥Bachelor’s degree	605 129	560 835 (535 889-585 290)	1.08 (1.03-1.13)	44 294 (19 838-69 239)	100
Total	2 960 001	2 434 496 (2 350 703-2 522 689)	1.22 (1.17-1.26)	525 505 (437 312-609 298)	100

Abbreviations: NA, not applicable; PI, prediction interval.

^a^ Excess mortality rates were estimated as the difference between observed and expected age-standardized mortality. Expected rates were forecasted from the baseline period (2006-2010). Observed and expected deaths were adjusted for age standardization. Totals by education were computed by aggregating expected and observed deaths over 1000 simulations from the models’ posterior distributions.

^b^ Values are NA when total excess deaths were negative.

## Discussion

Our findings suggest that 525 505 more deaths occurred in 2023 than would have been expected based on 2006-2010 mortality trends; 481 211 of these deaths were among noncollege graduates. The large number of excess deaths attributable to circulatory diseases underscores the ongoing association of cardiometabolic risk factors with US mortality trends, along with the social and structural conditions that shape these risks.^2^ External causes, particularly drug poisonings, were a major cause of excess deaths among male noncollege graduates in 2023, highlighting the ongoing role of deaths of despair in US mortality.^4^

This cross-sectional study has limitations. The composition of the educational categories evolved from 2006 to 2023 as more adults graduated college, meaning noncollege graduates may have represented a more disadvantaged population as the study progressed.^6^ Other limitations include the use of different data sources for death and population counts and the lack of disaggregation by more granular education categories. Future research is needed to quantify the role of specific mechanisms in producing the patterns documented here.

## Notes

Supplement 1.
eMethods
**eTable.** *International Statistical Classification of Diseases, Tenth Revision* (*ICD-10*) Codes Included in Cause-of-Death Categories
eReferences

Supplement 2.
Data Sharing Statement
