# Supplementary material for: Diverging Mortality Trends by Educational Attainment in the US
Source: JAMA Health Forum. 2025 Jun 13;6(6):e251647. doi: 10.1001/jamahealthforum.2025.1647 (PMC12166480; doi:10.1001/jamahealthforum.2025.1647)
Supplement: Supplement 1. — eMethods eTable. International Statistical Classification of Diseases, Tenth Revision (ICD-10) Codes Included in Cause-of-Death Categories eReferences [file jamahealthforum-e251647-s001.pdf]

Supplemental Online Content

Paglino E, Wrigley-Field E, Stokes AC. Diverging mortality trends by educational attainment in the US. *JAMA Health Forum*. 2025;6(6):e251647. doi:10.1001/jamahealthforum.2025.1647

eMethods

eReferences

eTable. *International Statistical Classification of Diseases*, *Tenth Revision* (*ICD-10*) Codes Included in Cause-of-Death Categories

This supplemental material has been provided by the authors to give readers additional information about their work.

© 2025 Paglino E et al. *JAMA Health Forum*.

eMethods

Data Sources

Cause-specific death counts: National Vital Statistics System Public Use Files 2006-2023.

Mid-year population estimates: American Community Survey 1-year Public Use Microdata Sample 2006-2023.

Alternative source for mortality rates: National Health Interview Survey Linked Mortality Files 1986-2018.

Estimating Death Counts and Mid-Year Populations

We obtained death counts by calendar year (2006-2023), sex, education (bachelor's degree or higher (BA) versus less than a BA), and five-year age groups (35-39, 40-44, 45-49 . . . 85+) from the Public Use Multiple Cause-of-Death files made available by the National Vital Statistics System. BA is used to refer to all four-year degrees. Deaths for which age was missing were assigned proportionally to valid age groups by year, census division, metro-nonmetro code, sex, and educational attainment. Similarly, deaths for which education was missing were assigned proportionally to the other education categories by year, census division, metro-nonmetro code, sex, and age. We restrict the sample to ages 35 or above, with ages 85+ as the open-ended age interval. Education was added to death certificates with the 1989 revision (NCHS 1991) but US states introduced it in different years. The reporting of education was again revised in 2003, moving from years of completed education to a highest degree attained. Again, many states implemented the change with delay and the transition was not completed until 2018. We follow the conversion strategy in previous NCHS studies to convert years of schooling completed to the highest degree attained.[1]

We used the 1-year American Community Survey files and the provided sampling weights to compute mid-year (July 1st) population estimates by year, sex, educational attainment, and 5-year age groups (35-39, 40-44, 45-49 . . . 85+). Years are limited to 2006-2023 because although ACS denominators are available from 2000, population estimates for 2000-2005 and those for 2006- show a discontinuity.

© 2025 Paglino E et al. *JAMA Health Forum*.

Causes of death

Our cause of death categories are derived from the CDC list of leading causes of death for 2019 [2], with simplified categories to ensure a large enough number of deaths in each category. **Table S1** reports the ICD-10 codes corresponding to our simplified categories.

Additional Estimates from the National Health Interview Survey Linked Mortality Files

Education reporting on death certificates is not consistent with the reporting of education in Census data sources or survey data[1,3] and can lead to incorrect conclusions about the magnitude and evolution of educational disparities in mortality.[4] The main alternative to estimating education-specific mortality rates is to use survey data, where education is self-reported, linked with death certificate data. A popular dataset to construct education-specific mortality rates, thanks to its large sample size, is the National Health Interview Survey Linked Mortality Files (NHIS-LMF), which has been widely used for this purpose.[4-11]

In this paper, we used data from the NHIS from 1986 to 2018 to estimate mortality rates by sex, educational attainment, and 5-year age group for three periods (2006-2009, 2010-2014, and 2015-2018. While education reporting in the NHIS-LMF is of higher quality than on death certificates, mortality estimates from the NHIS-LMF are consistently lower than those obtained in official life tables.[12,13] We adjust for this underestimation across all education groups at the national level by using age- and sex-specific mortality rates in each three-year period based on the NVSS-ACS estimates described above. This procedure is equivalent to raising or lowering sex-, and age-specific NHIS-LMF mortality rates to match those in the NVSS-ACS estimates, while preserving the educational gradient in the unadjusted NHIS data.

Adjusting NVSS-ACS Education-Specific Mortality Rates Using Adjusted NHIS Data

To correct for the inconsistencies in the reporting of educational attainment between NVSS and ACS data, we build on a previous adjustment procedure[14] to construct adjustment factors based on the adjusted NHIS-LMF mortality rates as follows. Denoting with $M_{NVSS,x,t,s,e}$ the death rate for age group $[x, x+5)$, period t (1: 2006-2009, 2: 2010-2014, 3: 2015-2018), sex s, and education category e computed from the NVSS-ACS data, and the equivalent rate computed from the NHIS-LMF as $M_{NHIS,x,t,s,e}$, the adjustment factor is:

$$A_{x,t,s,e} = \frac{M_{NHIS,x,t,s,e}}{M_{NVSS,x,t,s,e}}$$

© 2025 Paglino E et al. *JAMA Health Forum*.

This factor is applied to convert $M_{NVSS,x,t,s,e}$ into $M_{NHIS,x,t,s,e}$. For increased stability, we average the adjustment factors over the three periods, and we assume that the adjustment factors are equally valid across all causes of death. Denoting the averaged adjustment factors as $A_{x,s,e}$, our adjusted rates, now computed separately for each year y rather than for the multi-year periods t, and separately by cause c are then:

$$M_{NVSS\ ADJUSTED,x,y,s,e,c} = M_{NVSS,x,y,s,e,c} \cdot A_{x,s,e}$$

The effect of the adjustment process is to keep mortality trends by education as they appear in the NVSS rates but borrow educational gradients from the NHIS-LMF data. Together with the initial adjustment of NHIS rates to match the mortality level observed in NVSS data, this second adjustment should greatly increase the reliability of our estimates while maintaining two key advantages of using vital statistics data: 1) the entire population is captured, and 2) reliable estimates disaggregated by sex, education, single calendar year, and cause of death can be obtained.

Statistical Analysis

We denote with $D_{x,y,e}$ the number of deaths occurred in year y among those aged x to $x + 4$, with education level e, the corresponding exposures with $E_{x,y,e}$, and the corresponding mortality rate with $M_{x,y,e}$. We then compute age-standardized mortality rates as:

$$M_{STD,y,e} = \sum_{x} M_{x,y,e} \cdot c_x$$

Where c_x are age-specific population proportions from the 2000 US Standard Population. Denoting the entire population with educational attainment e in year y as $E_{y,e}$, we then compute the total number of deaths we would observe if the population in a given year and for a given education group had the same age-distribution as the standard population:

$$D_{STD,y,e} = M_{STD,y,e} \cdot E_{y,e}$$

Finally, our model is:

$$D_{STD,y,e} \sim NegativeBinomial(\mu_{STD,y,e} \cdot E_{y,e}, \theta)$$

$$log(\mu_{STD,y,e}) = \beta_e + s_e(y)$$

© 2025 Paglino E et al. *JAMA Health Forum*.

Where $\mu_{STD,y,e} \cdot E_{y,e}$ is the mean of the Negative Binomial distribution and θ is its overdispersion parameter. The model includes intercepts for educational attainment (β_e). Time is modeled with a smooth function $s_e(\cdot)$, which, as indicated by the e subscript, is allowed to differ by educational attainment. The smooth functions are expressed as deviations from the education-specific intercepts and constrained to sum to zero for identifiability. Smooth functions are defined as linear combinations of thin plate regression splines[15] with maximum basis dimension k set to 3 to avoid overfitting. The key assumption we are making is that the trend in the baseline period is smooth (no large year-to-year discontinuities) and then extend, linearly to future years with progressively widening confidence intervals. Models are fit with the *bam* function from the *mgcv* package[16] for the R programming language.

We fit separate models by sex and cause of death with an additional model for all-cause mortality. To reconcile cause-specific and all-cause estimates, we rescale expected deaths from the cause-specific models to sum to the expected deaths from the all-cause models.

When reporting expected and observed deaths in **Table**, we adjust deaths counts to account for the age-standardization procedure by multiplying standardized deaths counts by the correction factor:

$$a_{STD,y,e} = \frac{D_{y,e}}{D_{STD,y,e}}$$

Where $D_{y,e}$ is the observed number of deaths in year y for education group e and $D_{STD,y,e}$ is the age-standardized equivalent. If the population for a given year and group had exactly the same age-distribution as the standard population, these two numbers would be equivalent but there will generally be a small discrepancy. The correction we implement has the effect of converting observed age-standardized deaths into observed deaths and is also applied to expected deaths to make the two counts comparable.

We chose to model age-standardized mortality rates because when it comes to cause-specific and education-specific forecasting, age-specific rates can be quite unstable for ages at which mortality from a given cause is low. Especially given the rather long projection period, projecting age-specific rates occasionally led to implausible age distribution of deaths for future years. At the same time, crude mortality rates are not suitable for comparison across groups because differences in the age distribution will be a major source of confounding. After experimenting with different alternatives, we found that working with age-standardized rates provided both stability

© 2025 Paglino E et al. *JAMA Health Forum*.

and comparability. This comes at the cost of then having to de-standardize the rates to obtain estimates of excess deaths, but we feel this is a good compromise given the advantages.

Sensitivity Analysis

We performed three sensitivity checks to investigate the robustness of our results to different modeling and design choices. First, we tested using a more granular classification for educational attainment with five different categories: “less than high school”, “high school graduate”, “some college but no degree or associate’s degree”, “bachelor’s degree”, and “graduate or professional degree”. Second, we tested moving the threshold for the baseline period from 2010 to 2012. We also tested moving the threshold backward as well (to 2009) but this left us with too few baseline years to estimate the models reliably. Third, we tested increasing the basis dimension (k) for the smooth term on time in the GAM models. Note that in principle, fitting procedures for GAM models are capable of selecting the optimal complexity (in terms of effective degrees of freedom) for the smooth terms automatically, and the choice of k should only set the maximum complexity. However, with the limited number of data points in this study (only 5 with the 2006-2010 baseline), the estimates could be sensitive to different degrees of flexibility (which can be thought of as increasing the degree of a polynomial term).

Regarding the first sensitivity check, results were generally consistent with those obtained from the binary education categories (BA and no BA). In particular, mortality for the “bachelor’s degree” and the “graduate or professional degree” groups behave similarly and show moderate excess among males and lower than expected mortality among females. Similarly, mortality for the “less than high school”, “high school graduate”, and “some college but no degree or associate’s degree” displays slower-than-expected declines and thus positive excess mortality. The proportional differences between these three groups (absolute difference in the log scale) are roughly constant over time but the “some college but no degree or associate’s degree” group exhibits a larger proportional deviation from the expected rates.

Regarding the second sensitivity check, the results remained qualitatively similar. All groups excluding women with a BA showed higher than expected mortality. The main difference was for women, for whom the slowdown in mortality was already evident in 2011 and 2012, so that including these two years in the baseline period reduces the expected rate of mortality decline (particularly for women without a BA). Male mortality continued to show a decline consistent with late 2000s trends until 2012 so including 2011 and 2012 in the baseline period makes very little difference for the estimated excess mortality.

© 2025 Paglino E et al. *JAMA Health Forum*.

Regarding the third sensitivity check, estimates for all groups are insensitive to the additional flexibility allowed by setting k to 5. Women with a BA, continue to be the only group showing negative excess mortality, with other groups displaying higher than expected mortality.

Further information available

Underlying data, code, and additional visualizations are available in an OSF repository.

© 2025 Paglino E et al. *JAMA Health Forum*.

eReferences

1. Rostron B, Boies J, Arias E. Education reporting and classification on death certificates in the United States. *Vital Health Stat*. 2010;Series 2(151). Accessed December 7, 2023. https://pubmed.ncbi.nlm.nih.gov/25093685/
2. NCHS. Leading causes of death and number of deaths, by age: United States, 1980 and 2019. Published online 2021. https://www.cdc.gov/nchs/data/hus/2020-2021/LCODAge.pdf
3. Sorlie PD, Johnson NJ. Validity of Education Information on the Death Certificate. *Epidemiology*. 1996;7(4):437. Accessed March 12, 2024.
4. Hendi AS. Trends in Education-Specific Life Expectancy, Data Quality, and Shifting Education Distributions: A Note on Recent Research. *Demography*. 2017;54(3):1203-1213. doi:10.1007/s13524-017-0574-2
5. Cutler DM, Lange F, Meara E, Richards-Shubik S, Ruhm CJ. Rising educational gradients in mortality: The role of behavioral risk factors. *J Health Econ*. 2011;30(6):1174-1187. doi:10.1016/j.jhealeco.2011.06.009
6. Hendi AS. Trends in U.S. life expectancy gradients: the role of changing educational composition. *Int J Epidemiol*. 2015;44(3):946-955. doi:10.1093/ije/dyv062
7. Hummer RA, Lariscy JT. Educational Attainment and Adult Mortality. In: Rogers RG, Crimmins EM, eds. *International Handbook of Adult Mortality*. Springer Netherlands; 2011:241-261. doi:10.1007/978-90-481-9996-9_12
8. Krueger PM, Tran MK, Hummer RA, Chang VW. Mortality Attributable to Low Levels of Education in the United States. *PLOS ONE*. 2015;10(7):e0131809. doi:10.1371/journal.pone.0131809
9. Masters RK, Hummer RA, Powers DA. Educational Differences in U.S. Adult Mortality: A Cohort Perspective. *Am Sociol Rev*. 2012;77(4):548-572. doi:10.1177/0003122412451019
10. Montez JK, Berkman LF. Trends in the Educational Gradient of Mortality Among US Adults Aged 45 to 84 Years: Bringing Regional Context Into the Explanation. *Am J Public Health*. 2014;104(1):e82-e90. doi:10.2105/AJPH.2013.301526
11. Montez JK, Zajacova A. Trends in Mortality Risk by Education Level and Cause of Death Among US White Women From 1986 to 2006. *Am J Public Health*. 2013;103(3):473-479. doi:10.2105/AJPH.2012.301128
12. Case A, Deaton A. Life expectancy in adulthood is falling for those without a BA degree, but as educational gaps have widened, racial gaps have narrowed. *Proc Natl Acad Sci*. 2021;118(11):e2024777118. doi:10.1073/pnas.2024777118
13. Sasson I. Reply to Trends in Education-Specific Life Expectancy, Data Quality, and Shifting

© 2025 Paglino E et al. *JAMA Health Forum*.

Education Distributions: A Note on Recent Research. *Demography*. 2017;54(3):1215-1219. doi:10.1007/s13524-017-0583-1
14. Ho JY. The contribution of drug overdose to educational gradients in life expectancy in the United States, 1992-2011. Demography. 2017;54(3):1175-1202.
15. Wood SN. Thin Plate Regression Splines. *J R Stat Soc Ser B Stat Methodol*. 2003;65(1):95-114. doi:10.1111/1467-9868.00374
16. Wood S. mgcv: Mixed GAM Computation Vehicle with Automatic Smoothness Estimation. Published online December 21, 2023. Accessed September 29, 2024. https://cran.r-project.org/web/packages/mgcv/index.html

© 2025 Paglino E et al. *JAMA Health Forum*.

eTable. *International Statistical Classification of Diseases*, *Tenth Revision* (*ICD-10*) Codes Included in Cause-of-Death Categories

**Cause-of-Death Group**	**ICD-10 Codes**
Circulatory diseases	I00-I99
Diabetes	E10-E14
Malignant neoplasms	C00-C96
Drug poisonings	X40-44, X60-64, X85, and Y10-Y14
Other external	V01-Y89 excluding X40-44, X60-64, X85, and Y10-Y14
COVID-19	U071
All other causes	All ICD-10 codes not listed elsewhere

© 2025 Paglino E et al. *JAMA Health Forum*.
