# Supplementary material for: Diverging Mortality Trends by Educational Attainment in the US
Source: JAMA Health Forum. 2025 Jun 13;6(6):e251647. doi: 10.1001/jamahealthforum.2025.1647 (PMC12166480; doi:10.1001/jamahealthforum.2025.1647)
Supplement: Supplement 2. — Data Sharing Statement [file jamahealthforum-e251647-s002.pdf]

Data Sharing Statement

Paglino. Diverging Mortality Trends by Educational Attainment in the US. *JAMA Health Forum*. Published June 13, 2025. doi:10.1001/jamahealthforum.2025.1647

Data
Data available: Yes
Data types: Data (not involving human participants)
How to access data: All data and statistical code will be made available in an OSF repository (https://osf.io/vqbes/?view_only=8811837bd5824f99ad67f5334d214141) at the time of publication.
When available: With publication

Supporting Documents
Document types: Statistical/analytic code
How to access documents: All data and statistical code will be made available in an OSF repository (https://osf.io/vqbes/?view_only=8811837bd5824f99ad67f5334d214141) at the time of publication.
When available: With publication

Additional Information
Who can access the data: Anyone requesting the data
Types of analyses: For any purpose
Mechanisms of data availability: With investigator support
